# Brief Report on the Efficacy of Nivolumab in Patients With Previously Treated Advanced Large-Cell Neuroendocrine Cancer of the Lung

**DOI:** 10.1016/j.jtocrr.2020.100129

**Published:** 2020-12-10

**Authors:** Camille Agar, Margaux Geier, Guillaume Léveiller, Régine Lamy, Jean-Louis Bizec, Marie Tiercin, Cyril Bernier, Gilles Robinet, Hervé Léna, Charles Ricordel, Romain Corre

**Affiliations:** a Centre Hospitalier Universitaire de Rennes, Service de Pneumologie, Université de Rennes 1, Rennes, France; b Centre Hospitalier Régional Universitaire Morvan, Service d’Oncologie, Université de Bretagne Occidentale, Brest, France; c Centre Hospitalier Yves le Foll, Service de Pneumologie, Saint-Brieuc, France; d Centre Hospitalier Bretagne Sud, Service d’Oncologie, Lorient, France; e Centre Hospitalier Bretagne-Atlantique, Service de Pneumologie, Vannes, France; f Centre Hospitalier Saint Malo, Service de Pneumologie, Saint Malo, France; g Centre Hospitalier Rene Pleven, Service de Pneumologie, Dinan, France; h Institut National de la Santé et de la Recherche Médicale U1242, Chemistry Oncogenesis Stress and Signalling, Centres de Lutte Contre le Cancer Eugène Marquis, Rennes, France; i Centre Hospitalier Intercommunal de Cornouaille, Service de Pneumologie, Quimper, France

**Keywords:** Large-cell neuroendocrine cancer, Lung cancer, Immune checkpoint inhibitors, Nivolumab

## Abstract

**Introduction:**

The optimal management of large cell neuroendocrine cancer of the lung (LCNEC) is unclear, and data regarding anti–programmed cell death protein 1 (PD-1) antibodies are scarce. This study reports the clinical efficacy of a PD-1 inhibitor in patients with advanced LCNEC.

**Methods:**

All patients with stage III to IV LCNEC treated with at least one previous cycle of chemotherapy between January 1, 2015 and December 31, 2018 were reviewed retrospectively. Patients were divided into two groups depending on their exposure to nivolumab as second-line treatment or beyond. The primary objective was to assess nivolumab’s efficacy.

**Results:**

A total of 51 patients with advanced LCNEC from eight centers were analyzed, including 17 who received nivolumab. The PD-1 inhibitor was used as second-line treatment in 77% of cases, with a median number of eight doses (range: 1–62). After nivolumab treatment, the median overall survival was 12.1 months (95% confidence interval [CI]: 7.10–14.20). The objective response rate was 29.4% (95% CI: 10.3–56.0), and median progression-free survival was 3.9 months (95% CI: 1.68–7.17). The programmed death-ligand 1 status was unknown. There was no difference in the efficacy of first-line chemotherapy; the objective response rate was 23.5% (n = four of 17) in the nivolumab group versus 32.4% (n = 11 of 34) in the conventional treatment group, and progression-free survival was 3.5 months (95% CI: 1.7–4.4) versus 2.1 months (95% CI: 1.4–4.2), respectively.

**Conclusions:**

In a real-world setting, nivolumab seems to be an effective second-line treatment in patients with advanced LCNEC. Large prospective studies in this setting are still required.

## Introduction

Large cell neuroendocrine cancer of the lung (LCNEC) is rare. Its histologic features include large cells, a high mitotic rate, extensive necrosis, and a neuroendocrine growth pattern. In the WHO classification in 2015, these carcinomas belong to the group of neuroendocrine tumors.^1^ At the metastatic stage, this type of cancer is aggressive with a poor prognosis—the median survival ranges from 7 to 12 months, depending on the study.^2^ There is currently no consensus on the best treatment for advanced LCNEC,^3^ but a combination of platinum-etoposide is the most frequently used as first-line therapy.

The past decade has been marked by the development of immune checkpoint inhibitors (ICIs) for lung cancer. Nivolumab, an anti–programmed cell death protein 1 antibody, is currently used as second-line therapy for advanced NSCLC with a 9% improvement in 3-year survival compared with docetaxel.^4^^,^^5^ This option has rarely been explored for LCNEC. Nevertheless, some cases of response to immunotherapy have been reported in the literature. The level of expression of the programmed death-ligand 1 (PD-L1) receptor on the surface of tumor cells, a biomarker of the response to ICI, seems to be the most elevated in neuroendocrine tumors.^6^ The perspectives offered by this treatment are interesting, taking into account the limited therapeutic options for this rare form of cancer.

We carried out a multicenter retrospective study of patients with LCNEC who received nivolumab and analyzed its effectiveness.

## Materials and Methods

### Study Design and Patients

This multicenter retrospective study included all cases of advanced LCNEC (stages IIIB–IV) diagnosed in eight hospital centers in the Brittany region of France between January 2015 and December 2018. Eligible patients were identified from the histologic databases. In centers where histologic databases were not available, databases that included all patients with lung cancer treated with platinum-etoposide chemotherapy were used. Diagnosis of LCNEC was performed by pathologists in each center at the time the disease was discovered. The disease could be either metastatic or relapsing after local treatment. All patients should have received at least one previous course of chemotherapy without concomitant radiotherapy.

### Study Treatment

The principal objective was to determine the overall survival (OS) of the patients treated with nivolumab and who received at least one dose, calculated from the date of the first nivolumab injection. The secondary objectives were to determine progression-free survival (PFS) and objective response rate (ORR), determined by the investigators in each center according to Response Evaluation Criteria in Solid Tumors version 1.1. Patients treated with nivolumab were compared for characteristics and response to prenivolumab systemic treatments with other patients with LNCEC treated with conventional second-line therapy during the same period. This noninterventional study was approved by the ethics committee (notice number 19.108) of the Centre Hospitalier Universitaire Rennes, Rennes, France, and by the Commission Nationale Informatique et Liberté in accordance with French law. Informed consent was obtained through a nonopposition letter sent to participants.

## Results

### Characteristics of the Study Population

**Supplementary Figure 1:**
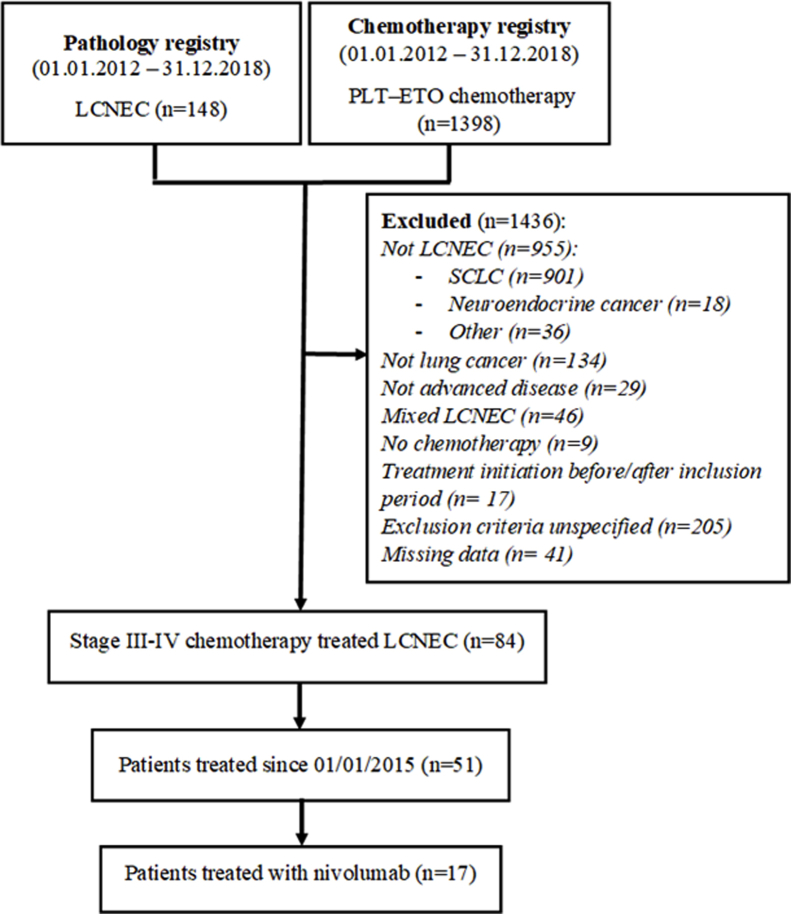


A total of 51 patients were included in the study, including 17 who were treated with nivolumab (Supplementary Fig. 1). The clinical characteristics of the patients at the time of diagnosis were similar in the two groups (Table 1), although performance status seems to be better, the number of metastatic sites was lower, and the number of lines was higher in the nivolumab group than the conventional therapy group. PD-L1 status was not available for all patients.

**Table 1:** Baseline Characteristics of the Patients With Pulmonary LCNEC

Characteristics	Total (n = 51)	Nivolumab (n = 17)	Conventional Treatment (n = 34)	*p* Value
Age, y, median (range)	59 (38–82)	59 (42–79)	59 (38–82)	0.8155
Sex, male, n (%)	41 (80)	12 (71)	29 (85)	0.2700
Smoking status, n (%)	49 (2)^a^	17 (0)^a^	32 (2)^a^	1.0000
Current/former smoker	48 (98)	17 (100)	31 (97)	
ECOG PS, n (%)	43 (8)^a^	12 (5)^a^	31 (3)^a^	0.1630
>1	7 (16%)	0 (0%)	7 (23%)	
Weight loss, n (%)	44 (7)^a^	13 (4)^a^	31 (3)^a^	1.0000
>10%	12 (27)	3 (23)	9 (29)	
Stage, n (%)				
III	4 (8)	1 (6)	3 (9)	1.0000
IV	47 (92)	16 (94)	31 (91)	
De novo, n (%)	43 (84)	13 (77)	30 (88)	0.4156
Recurrent, n (%)	8 (16)	4 (24)	4 (12)	
Number of metastatic organs, n (%)				
<3	40 (79)	16 (94)	24 (71)	0.0751
≥3	11 (22)	1 (6)	10 (29)	
Brain metastasis, n (%)	10 (20)	2 (12)	8 (24)	0.4632
Number of courses, median (range)	2 (1–6)	3 (2–6)	1 (1–4)	<0.0001

ECOG, *Eastern Cooperative Oncology Group*; LCNEC, large-cell neuroendocrine cancer of the lung; PS, performance status.

a Missing date.

### Efficacy of Nivolumab

**Figure 1:**
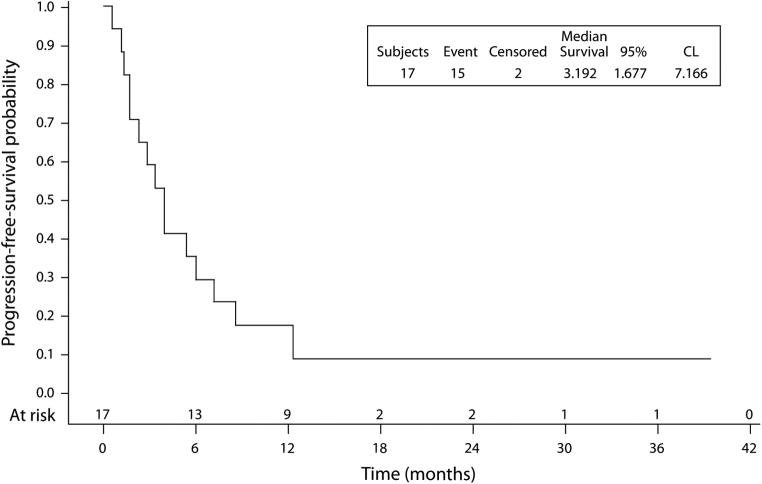
PFS from nivolumab initiation. CL, confidence level; PFS, progression-free survival.

**Figure 2:**
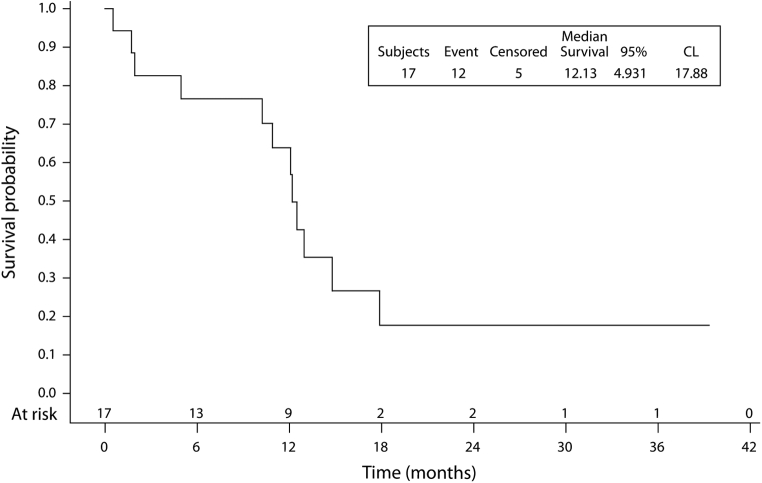
Overall survival from nivolumab initiation. CL, confidence level.

In the group treated with nivolumab, immunotherapy was a second-line treatment in 77% of cases and as a third-line in 23%. The median number of cycles of nivolumab received by the patients was eight (range: 1‒62). The median OS from the introduction of nivolumab was 12.1 months (95% CI: 7.10–14.20) (Fig. 1). The survival rate was 76.5% (95% confidence interval [CI]: 48.8–90.4) at 6 months, 63.7% (95% CI: 36.3–81.9) at 12 months and 17.7% (95% CI: 3.1–42.0) at 18 months. The median PFS was 3.9 months (95% CI: 1.68–7.17) (Fig. 2); PFS was 58.8% (95% CI: 32.5–77.8) at 3 months, 29.4% (95% CI: 10.7–51.1) at 6 months, and 17.6% (95% CI: 4.3–38.3) at 12 months.

The ORR was 29.4% (95% CI: 10.3–56), and the disease control rate was 58.8% (95% CI: 32.9–81.6). The median duration of response was 6.5 months (Table 2). No evaluation could be carried out on two patients because of early death, and 30% of patients had disease progression at their first evaluation. At the end of the study period, two patients had not progressed with nivolumab after 27 and 62 cycles, respectively, and six patients had received more than 6 months of treatment.

**Table 2:** Clinical Response to Nivolumab

Response	Total (n = 17)	
	n	% (95% CI)
ORR	5	29.4 (10.3–56.0)
Complete response	0	0
Partial response	5	29.4
Stable disease	5	29.4
Controlled disease	10	58.8 (32.9–81.6)
Progressive disease	5	29.4
Not assessable	2	11.8
Duration of response (mo), median	6.5	(3.68–8.84)

CI, confidence interval; ORR, objective response rate.

### Treatment Before nivolumab

Before ICI, almost all patients received a platinum-based first-line chemotherapy regimen, usually a combination of platinum and etoposide (71% of cases). Only 18% of patients had responded to treatment preceding immunotherapy. Nearly half of the patients received previous radiotherapy, either curative or palliative.

There was no difference in the efficacy of first-line treatment between the two groups (Table 3). The ORR of first-line treatment was 23.5% (n = four of 17) in the nivolumab group versus 32.4% (n = 11 of 34) in the conventional treatment group. PFS was 3.5 months (95% CI: 1.7–4.4) versus 2.1 months (95% CI: 1.4–4.2), respectively.

**Table 3:** First-Line Chemotherapy

Group	Total (n = 49)	Nivolumab (n = 17)	Conventional Treatment (n = 32^a^)	*p* Value
Type of chemotherapy, n (%)				0.0406
Plt-Eto	42 (86)	12 (71)	30 (94)	
Other	7 (14)	5 (29)	2 (6)	
Reason for stopping treatment, n (%)				0.1551
Treatment completed	8 (16)	1 (6)	7 (21)	
Disease progression	21 (41)	10 (59)	11 (32)	
Toxicity	6 (12)	2 (12)	4 (12)	
Medical decision	10 (20)	3 (18)	7 (21)	
Patient’s preference	1 (2)	1 (6)	0 (0)	
Death	5 (10)	0 (0)	5 (15)	
Efficacy				
ORR (%) [95% CI]	(29.4) [17.5–43.8]	(23.5) [6.8-–49.9]	(32.4) [17.4–50.5]	0.5145
PFS (mo), median [95% CI]	—	3.5 [1.7–4.4]	2.1 [1.4–4.2]	0.5427

CI, confidence interval; Eto, etoposide; ORR, objective response rate; PFS, progression-free survival; Plt, platinum.

a Missing data.

### Treatment After Nivolumab

A total of 10 patients received systemic treatment after progression with nivolumab. Taxane-based monotherapy was the most common first-line therapy after progression (n = 5). Other options were the following: (1) rechallenge with platinum-etoposide (n = 2); (2) monochemotherapy with gemcitabine (n = 1); or (3) pemetrexed (n = 1), with one patient included in an early phase trial. There were no cases exhibiting response to chemotherapy after immunotherapy, irrespective of the protocol used (missing data, n = 2).

## Discussion

To our knowledge, this is the largest study to date that describes patients who received immunotherapy for advanced LCNEC after first-line chemotherapy. The median OS was 12.1 months, which is comparable with patients with advanced NSCLC in the CheckMate-017 and 057 trials (9.2 mo and 12.2 mo, respectively).^4^^,^^5^ Patients with LCNEC are good candidates for ICI therapy with 29.4% ORR (versus 20% and 19%, respectively) and comparable median PFS of 3.5 months (versus 3.5 mo and 2.3 mo, respectively). These results seem better than the usual second-line therapies for LCNEC, in which available data are rare. Chemotherapy protocols are not standardized and may be similar to those used in SCLC or NSCLC, with response rates of less than 20%.^7^^,^^8^

Advanced LCNEC is often incorrectly related to SCLC, the latter of which is usually very chemosensitive and in which nivolumab gave disappointing results as second-line therapy (CheckMate-331), with low PD-L1 expression. According to retrospective studies, the rate of PD-L1–positive LCNEC is estimated to be the highest of all neuroendocrine tumors, which is between 10% and 27%.^9^^,^^10^ In our study, PD-L1 levels were not available for the patients because the marketing authorization for nivolumab is independent of the level of PD-L1 expression. A few cases of response to immunotherapy have already been described in the literature.^6^^,^^11^ A recent retrospective French study analyzed 10 patients with stage IIIB to IV LCNEC treated with immunotherapy after platinum-based chemotherapy. The patients received a mean of 16 cycles, and the median PFS was 57 weeks.^12^ Our study confirmed these promising results.

The clinical profile of all the patients is a classic case of LCNEC: a predominance of young men and smokers.^3^ The use of platinum-etoposide combination as first-line treatment is in agreement with current recommendations, although the chemosensitivity of LCNEC is inferior to what is observed in SCLC, as illustrated by the modest response rate to chemotherapy (29.4%) in our study.^2^^,^^13^ In the nivolumab group, patients who had a better prognosis could have been selected. There is a tendency for better performance status and fewer metastatic sites, particularly brain metastases. The response rates and PFS after first-line chemotherapy were similar in the two groups, but only 47% of patients in the conventional treatment group received second-line treatment.

This small retrospective study has several limitations. First, there was no centralized review of histologic data. It is known that LCNEC can be difficult to diagnose.^14^ In our study, the diagnosis of LCNEC was only made secondarily for five patients, which could explain why they received first-line chemotherapy other than platinum-etoposide. There is no exhaustive register of cases of LCNEC, and a large search strategy was therefore performed. Second, TP53 or RB1 tumoral status was not assessed in this study. As the molecular heterogeneity of LCNEC has been reported to predict systemic treatment efficacy,^15^ future trials evaluating immunotherapy in this disease should encompass exhaustive genomic profiling.

Finally, in this real-world study, the evaluation of the response to treatment was assessed by the investigator in each center without blinded independent central review. However, this retrospective study has the advantage of reporting the results reflecting current practice involving this underexplored disease.

In conclusion, this report highlights a promising activity of nivolumab in patients with LCNEC, exhibiting a high level of tumor response and prolonged OS as second-line treatment or beyond. These results are encouraging in this setting, in which the therapeutic options are limited. However, these results should be confirmed, and trials testing the combination of anti–PD-L1 or anti-PD-1 and chemotherapy as first-line therapy are now required.

## References

[1] Travis W.D., Brambilla E., Nicholson A.G. (2015). The 2015 World Health Organization classification of lung tumors: impact of genetic, clinical and radiologic advances since the 2004 classification. J Thorac Oncol.

[2] Naidoo J., Santos-Zabala M.L., Iyriboz T. (2016). Large cell neuroendocrine carcinoma of the lung: clinico-pathologic features, treatment, and outcomes. Clin Lung Cancer.

[3] Fasano M., Della Corte C.M., Papaccio F., Ciardiello F., Morgillo F. (2015). Pulmonary large-cell neuroendocrine carcinoma: from epidemiology to therapy. J Thorac Oncol.

[4] Borghaei H., Paz-Ares L., Horn L. (2015). Nivolumab versus docetaxel in advanced nonsquamous non–small-cell lung cancer. N Engl J Med.

[5] Brahmer J., Reckamp K.L., Baas P. (2015). Nivolumab versus docetaxel in advanced squamous-cell non–small-cell lung cancer. N Engl J Med.

[6] Daido W., Yamasaki M., Saito N. (2017). Effectiveness of nivolumab in large-cell neuroendocrine carcinoma of the lung - a report of two cases. Gan To Kagaku Ryoho.

[7] Shimada Y., Niho S., Ishii G. (2012). Clinical features of unresectable high-grade lung neuroendocrine carcinoma diagnosed using biopsy specimens. Lung Cancer.

[8] Yoshida H., Sekine I., Tsuta K. (2011). Amrubicin monotherapy for patients with previously treated advanced large-cell neuroendocrine carcinoma of the lung. Jpn J Clin Oncol.

[9] Tsuruoka K., Horinouchi H., Goto Y. (2017). PD-L1 expression in neuroendocrine tumors of the lung. Lung Cancer.

[10] Inamura K., Yokouchi Y., Kobayashi M. (2017). Relationship of tumor PD-L1 (CD274) expression with lower mortality in lung high-grade neuroendocrine tumor. Cancer Med.

[11] Mauclet C., Duplaquet F., Pirard L. (2019). Complete tumor response of a locally advanced lung large-cell neuroendocrine carcinoma after palliative thoracic radiotherapy and immunotherapy with nivolumab. Lung Cancer.

[12] Levra M.G., Mazieres J., Valette C.A. (2017). P1.07-012 efficacy of immune checkpoint inhibitors in large cell neuroendocrine lung cancer: results from a French retrospective cohort. J Thorac Oncol.

[13] Masters G.A., Temin S., Azzoli C.G. (2015). Systemic therapy for Stage IV non–small-cell lung cancer: American Society of Clinical Oncology clinical practice guideline update. J Clin Oncol.

[14] Pelosi G., Barbareschi M., Cavazza A., Graziano P., Rossi G., Papotti M. (2015). Large cell carcinoma of the lung: a tumor in search of an author. A clinically oriented critical reappraisal. Lung Cancer.

[15] Derks J.L., Leblay N., Thunnissen E. (2018). Molecular subtypes of pulmonary large-cell neuroendocrine carcinoma predict chemotherapy treatment outcome. Clin Cancer Res.

